# Rise of ‘Lonely’ Consumers in the Post-COVID-19 Era: A Synthesised Review on Psychological, Commercial and Social Implications

**DOI:** 10.3390/ijerph18020404

**Published:** 2021-01-06

**Authors:** Xueqin Wang, Yiik Diew Wong, Kum Fai Yuen

**Affiliations:** 1 Department of International Logistics, Chung-Ang University, Seoul 06974, Korea; xueqinwang@cau.ac.kr; 2 School of Civil and Environmental Engineering, Nanyang Technological University, Singapore 639798, Singapore; CYDWONG@ntu.edu.sg

**Keywords:** lonely consumers, social exclusion/isolation, literature review, COVID-19, social distancing, co-occurrence network analysis

## Abstract

Loneliness is a pervasive problem recognised as a serious social issue, and the prevailing COVID-19 pandemic has exacerbated loneliness to greater prominence and concern. We expect a rise of a massive group of ‘lonely’ consumers who are deeply entrenched in the social isolation caused by COVID-19. There is an urgent need to revisit the phenomenon of lonely consumers to better prepare academic researchers, public policy makers and commercial managers in the post-COVID-19 era. Thus, this study conducts a synthesised review on past studies of lonely consumers. Based on an inductive analysis of 56 articles, 74 key themes are identified. These key themes are further categorised into five major clusters by way of a co-occurrence network analysis. Respectively, the five clusters address the psychological implications related to the *dynamics between nonhuman attachment and consumers’ loneliness*, the commercial implications related to the *paradoxical motivations of affiliation and self-affirmation in product selection and the dual information processing mechanism in response to advertisement appeals*, and the social implications related to *consumers’ well-being in an ageing society* and the *anthropomorphic companionship in a virtual world*. A list of research questions is proposed that concludes the review study.

## 1. Introduction

Loneliness is a pervasive problem in modern societies [1]. Even before the COVID-19 pandemic, we have witnessed an emerging trend of individualised lifestyles, by choice or by circumstance, due to changing socio-demographics such as decreased household size, delayed marriages and longer life span coupled with increased relocation tendency (e.g., education and career requirements) [2,3]. Adolescents striving for personal identity and retirees lacking social companions both experience loneliness. Working adults who pursue careers away from their families and friends in large cities may feel lonely, whereas consumers who live in suburban areas may feel socially isolated due to a lack of access to social facilities and resources. Regardless of age and living environments, loneliness may be experienced by every consumer though in quite different manners. The recent advancement in communication technologies might come as a solution to the prevalent loneliness, yet constant connectivity via social media and personal mobile devices does not guarantee meaningful interactions [4]. Research has shown that people now have fewer quality social relationships, despite the increased quantity of online interactions [5]. Consumers end up with more, but weaker, connections that are technology-mediated and commensurately fewer strong ties that are essential to foster a sense of belongingness [6,7].

Although the state of the prevailing loneliness is already being recognised as a serious social issue, COVID-19 has exacerbated loneliness to greater prominence and concern. The COVID-19 pandemic hit the world in early 2020, and many countries are still facing serious health crisis when the current research is conducted (i.e., December 2020). With national practices of social distancing and lockdown at varying levels across the economies, consumers in some communities are advised to refrain from all non-essential social activities and learn to adjust to an ‘in-home everything’ lifestyle by working, dining and shopping at home [8]. Although these practices are temporary, prolonged social isolation may have long-lasting impacts on consumers. In particular, the overwhelming loneliness associated with in-home lifestyle is felt by millions of people [9,10]. Consumers who live alone, the elderly and those with pre-existing mental illness are particularly vulnerable [11,12]. Researchers have recently conducted many studies in different geographical contexts, and the large-scale polls all indicated that consumers are struggling to cope with pandemic-induced loneliness [13,14,15,16].

Against this background, we expect a rise of a massive group of ‘lonely’ consumers who are deeply entrenched in the social isolation kindled by the pandemic. Importantly, consumption activities are infused with rich social elements that make loneliness perception an especially salient consideration concerning consumers’ consumption behaviours. For example, shopping is not only about obtaining physical products but also provides enjoyable and socialising experiences that create happiness and enhance well-being [17]. Other examples include experiential consumption activities, such as dining and leisure travelling [18]. This is in line with the classical conceptualisation of consumption activities [19,20]. Indeed, consumption has long been recognised as social activities where consumers demonstrate their distinctiveness while seeking for recognition from important others. As a consequence, perceived loneliness may alter consumers’ mentality and thereby modify consumers’ consumption behaviours, which create theoretical and practical implications. Thus, there is an urgent need to revisit the phenomenon of lonely consumers to better prepare academic researchers, public policymakers and commercial managers in the post-pandemic era. To this end, this study conducts a synthesised review on past studies of lonely consumers. By doing so, we contribute to the literature by providing a timely answer to the question ‘How can loneliness/social isolation shape consumption behaviours in the post-pandemic era?’

More especially, based on an inductive analysis of 56 articles, 74 key themes are identified. These key themes are further converged into five major clusters through a co-occurrence network analysis pointing to psychological, commercial and social implications that revolve around consumers’ fundamental motivation for affiliation/belongingness.

The remainder of this manuscript is structured as follows. Firstly, Section 2 elaborates the review method, which details the three-step data collection process and data analysis techniques (i.e., inductive content analysis and co-occurrence network analysis). Next, Section 3 reports the main review findings containing the five thematic clusters. These five clusters address the psychological implications related to the dynamics between attachment to nonhuman and consumers’ loneliness (Section 3.1), the commercial implications related to the paradoxical motivations of affiliation and self-affirmation in product selection (Section 3.2) and the dual information processing mechanism in response to advertisement appeals (Section 3.3). Moreover, the social implications related to consumers’ well-being in ageing society (Section 3.4) and the anthropomorphic companionship in the virtual world (Section 3.5) are addressed. Lastly, to conclude the review study, Section 4 proposes a list of future research questions and presents the theoretical and practical implications.

## 2. Method

**Figure 1:**
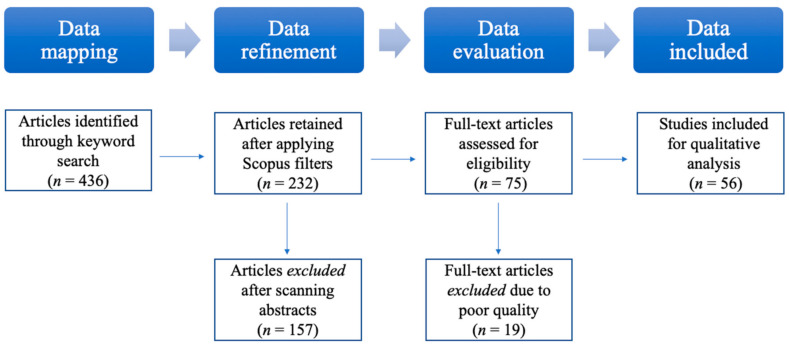
Research method.

This review study aims to locate and evaluate research articles related to lonely consumers so that synthesised insights can be created and future research agenda can be proposed. To ensure comprehensive coverage of peer-reviewed journal platforms, we use Scopus of Elsevier as the search database for reliable and updated journal articles [21]. The data collection process consists of three steps, namely, data mapping, data *refinement* and *data evaluation* [22]. A total of 56 articles are qualified after applying the three steps. The remainder of this section elaborates the three-step data collection process. Figure 1 provides an illustration of the data collection method.

### 2.1. Data Mapping

This step involves constructing appropriate keywords and keyword structures. We started with a straightforward keyword structure ‘lonely consumers’, which is directly relevant to the current study. Upon applying the keyword search, an initial list of results was generated. Thereafter, additional keywords were identified by scanning through the initial results (e.g., titles, authors’ keywords and abstracts), which were then incorporated into our keyword structure. Through several rounds of trial and error, a finalised keyword structure was devised.

Notably, the keyword ‘social exclusion’ appeared frequently during the trial and error stage. A brief examination on the related research revealed various connotations regarding social exclusion, which included a lack of resources to participate in social activities (e.g., due to poverty, disabilities or living in rural areas) [23,24]; being ignored or rejected in social settings [6,25]; and living in isolation voluntarily or involuntarily [26,27]. Here, although ‘social exclusion’ is not strictly a synonym to ‘loneliness’, life scenarios of ‘social exclusion’ are often associated with the mental state of ‘loneliness’. ‘Social exclusion’ is also an appropriate term to describe the current lifestyle of global consumers under the influence of COVID-19. Thus, we included ‘social exclusion’ as an alternative term to ‘loneliness’ in the keyword structure. In addition, the term ‘shoppers′ was identified as a common synonym to ‘consumers’. Therefore, the following finalised keyword structure was applied for keyword search in Scopus: (consumer * OR shopper *) AND (lonel * OR ‘social * exclu *’). A total of 436 publications were generated including journal articles, conference papers and book chapters.

### 2.2. Data Refinement

This step identifies the most relevant and reliable research among the 436 publications. To start with, we included only peer-reviewed journal articles because the research credibility was maintained by the review process. In addition, the subject area of ‘medicine’ was excluded because it contained a large stream of clinical research that did not apply to general consumers. Accordingly, applying the ‘document type’ and ‘subject area’ filters on Scopus resulted in a list of 232 journal articles. No further temporal or territorial criteria were used in the search.

Furthermore, the retained article titles and abstracts were assessed to determine their relevancy to the current study. In particular, the research objective statements were analysed to see whether the concept of ‘lonely consumer’ was a central theme of the respective articles. When the research’s key objectives cannot be extracted from the titles and abstracts, the conclusion sections were also read to shed more light. Only those articles that held ‘lonely consumers’ as a central theme to the entire research were included for further analysis. The refinement process obtained a total of 75 journal articles.

### 2.3. Data Evaluation

During the data evaluation step, all the retained 75 articles were read in their entirety. As authors to the review study, it is our responsibility to select only ‘quality’ papers for further review. Upon finishing reading each article, a subjective judgement was made as to whether to include it in the final review list. Accordingly, 19 articles were disqualified for further review due to unclear presentation, insufficient implications or inadequate communication. As such, a final list of 56 journal articles was created (see Appendix A for the summary).

Based on the finalised list of papers, inductive analysis and co-occurrence network analysis were performed, which are further discussed in Section 2.3.1 and Section 2.3.2, respectively.

#### 2.3.1. Inductive Analysis: Identification of Key Themes

A qualitative inductive analysis was conducted based on the 56 journal articles, where the key themes of each article were identified, compiled and sorted [28]. The analysis requires researchers to analyse the text contents with an open mind and identify meaningful subjects, keywords and phrases that can lead to conclusive insights. In other words, upon reading the selected articles, the researchers establish an overall understanding of the studies and propose key phrases as an indication of the themes that may emerge. Wherever and whenever possible, similar phrases are combined and condensed to reveal the key themes related to the concept of lonely consumers.

For example, key phrases such as *belongingness*, *affiliation* and *social inclusion* appeared in several studies, which all connoted similar meanings. Thus, they were condensed to form the theme of *affiliation motivation*. In a similar vein, the key theme *marketplace relationships* emerged from compiling several key phrases identified from the literature, such as *interaction with salesperson, consumer–brand interaction*, and *parasocial interaction with TV shopping hosts*. The analysis was primarily conducted by the lead author, who devoted a complete period on the analysis with minimal external distractions. The purpose was to ensure the same evaluation criteria were consistently applied throughout the data analysis. Whenever in doubt, the co-authors who are experts in the research field, were consulted and their opinions were taken into considerations to form the final decision. By doing so, 74 key themes emerged, which are listed in Appendix A. Table 1 provides some brief descriptions of the most frequently identified themes.

#### 2.3.2. Co-Occurrence Network Analysis: Formation of Theme Clusters

The identified key themes serve as input for the co-occurrence network analysis. The software VOSviewer, a computer programme that is specially designed to analyse bibliometric data, is used to perform the analysis [29]. It is particularly powerful in creating co-occurrence networks that are easy to interpret. By inputting the key themes, the connection strength of each theme with the rest is calculated, and a co-occurrence network is then created based on the most connected themes. To interpret the generated network, each theme is represented by a circle, and each circle is assigned to a coloured cluster. The size of the circle shows the occurrence frequency of the theme (e.g., the larger is the circle, the more frequent is the theme). Moreover, the thickness of the line connecting the two themes indicates their co-occurrence strength (e.g., the thicker the line, the stronger the co-occurrence). Our analysis produced a five-cluster network that reveals the five interconnected yet distinct implications of the extant literature on lonely consumers. The five-cluster network is elaborated in Section 3.

## 3. Lonely Consumers: Psychological, Commercial and Social Implications

**Figure 2:**
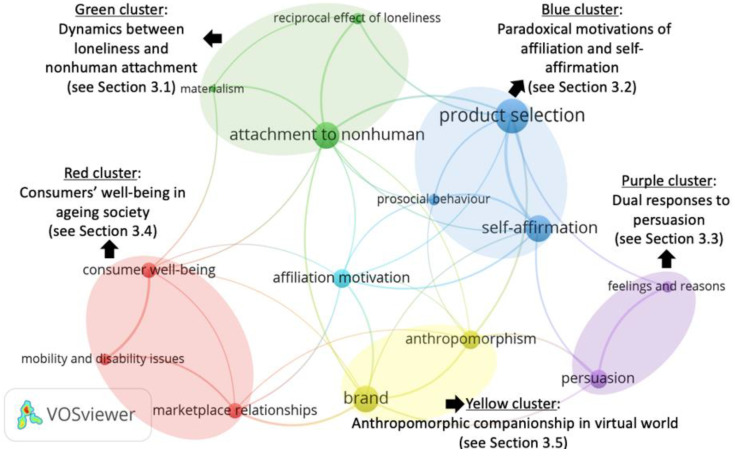
The co-occurrence network analysis of key themes.

The co-occurrence network is shown in Figure 2. The theme *affiliation motivation* emerges as a central theme that links the five coloured clusters. The green cluster reveals the *psychological* coping mechanism (e.g., compensatory attachment to nonhuman agents) that is fundamental to understanding lonely consumers (Section 3.1); the blue and purple clusters address the loneliness-induced consumption patterns that create commercial implications (Section 3.2 and Section 3.3); the red and lemon clusters centre on consumers’ well-being with marketplace companionship, thereby generating social implications (Section 3.4 and Section 3.5).

### 3.1. Psychological Dynamics between Loneliness and Nonhuman Attachment (Green Cluster)

This cluster contains three key themes, namely, *attachment to nonhuman*, *materialism* and *reciprocal effect of loneliness*. Linked to the central theme of *affiliation motivation*, this cluster addresses consumers’ psychological coping mechanism of loneliness when real social interactions are absent [30]. More specifically, it points to lonely consumers’ development of materialism and attachment to nonhuman agents (e.g., anthropomorphic product, virtual world and consumption behaviours) that potentially replace the need of social relationships [31,32,33]. However, our review suggests that seeking alternative social assurance from material possessions or other nonhuman agents may reinforce consumers’ isolation, which makes consumers feel even lonelier, signalling a reciprocal effect of loneliness [34,35].

This cluster of studies is built on the assumption that people have a basic motivation for affiliation, which is alternatively termed as relatedness, social connection, belongingness and attachment [34,36]. The affiliation motivation deeply resides in human survival instinct from an evolutionary perspective [30]. Although experiencing affiliation leads to life satisfaction, threats to social belongingness trigger primal responses that typically take the form of reconnection with other human beings [30]. However, when interactions with the real-world human beings are not directly available due to a lack of social skills or practice of social distancing, consumers tend to develop a compensatory mechanism to achieve a psychological balance. According to attachment theory, when failing to establish primary attachments with a human caretaker, people direct their reliance to material agents as secondary attachments (i.e., a compensation) [34]. In this regard, our identified literature suggests consumers’ attachment to nonhuman agents as a fundamental compensatory mechanism [37,38,39].

For example, Duclos, Wan and Jiang [37] found that consumers treat money and wealth as a substitute for popularity. In the absence of social support, consumers acutely take riskier financial decisions or even participate in gambling, which are perceived to be potentially more lucrative. They attempt to restore what they want out of the social system by emphasising the instrumentality value of money [37]. In a similar vein, Mead, Baumeister, Stillman, Rawn and Vohs [33] suggested that consumers use the actions of spending and consumption to strategically seek for affiliation. When in a desperate need of social connection, consumers spend on products that are favoured by a peer or symbolise a group membership even when illegal products are concerned (e.g., drugs). Such spending and consumption behaviours are considered socially lucrative by lonely consumers [33]. In a more vivid example, the anthropomorphic products, which are products with human-like features, are found to replace consumers’ need for interactions with real human beings [30]; by positioning the anthropomorphic products as companion pals, the authors suggested that consumers derive a sense of social assurance from the anthropomorphic products similarly with that of real-world social interactions.

Related to consumers’ attachment to nonhuman agents, the concept of materialism is also frequently examined. The former refers to consumers’ general reliance on nonhuman as a replacement for social interactions, whereas materialism involves consumers’ attachment to acquiring and owning material possessions, which is a much more restricted concept [34,40]. Similar to nonhuman agents, material substitutes compensate consumers’ need for belongingness, yet they are less anxiety-provoking and constantly available, which offer simple assurance and comfort to lonely consumers [34]. Empirical evidence also links materialism with consumers’ loneliness and social affiliation deficits [40].

More recently, with the rapid expansion of communication technologies, consumers’ unfulfilled connection needs find a convenient outlet in the virtual world [38]. Research shows that consumers develop an addiction to the Internet and attachment to social media when experiencing social isolation or loneliness, which may lead to compulsive buying behaviours and reduce consumers’ well-being [32,38]. Furthermore, consumers’ attachment to the virtual world is found to discourage them to reach out to the real world and, hence, reduces the quality of face-to-face interactions [30,38]. As encapsulated in the term ‘phubbed’ (i.e., phone snubbed), the real-world social interactions are constantly disrupted by people checking their phones and ignoring the interacting actors [38]. It creates the so-called ‘present-absent’ paradox where people are physically present for social interactions but mentally preoccupied elsewhere because of checking social media updates, replying instant messages and answering phone calls [38]. Ironically, as commented by David and Roberts [38] and Mourey, Olson and Yoon [30], the technologies which are designed to overcome physical communication barriers have created social isolation and loneliness in technology-mediated relationships. Indeed, consumers’ attachment to the virtual world, which is supposed to alleviate loneliness feelings, has made them even lonelier.

Our review study seems to suggest the dynamic between consumers’ attachment to nonhuman agents (including material possessions, i.e., materialism) and their loneliness [34,35,38]. In other words, the loneliness triggered by affiliation motivation causes consumers’ attachment to nonhuman agents, yet the nonhuman agents ‘crowd out’ the real social relationships, which in turn worsen consumers’ loneliness and social isolation. Such a reciprocal relationship is supported in both technology-mediated and conventional consumption contexts. For example, David and Roberts [38] study reveals that consumers who are ‘phubbed’ redirect their attention to social media and consequently disengage with social interactions. Within a conventional context, Pieters [34] reported the bidirectional dynamics between materialism and loneliness in a six-year longitudinal study. His findings suggest that loneliness contributes to materialism, and materialism reinforces loneliness to a weaker extent. These studies seem to point to a vicious cycle between consumers’ loneliness and attachment to nonhuman agents, in a way that the nonhuman agents which are treated as a ‘cure’ for loneliness become ‘the very obstacle that derail[s] typical compensatory behaviours designed to restore social connection’ [30].

However, some researchers argued otherwise for a potentially virtuous dynamic. Focusing on different subtypes of materialism, Pieters [34] found that valuing possessions as a *measure* of success (subtype 1) and as a *medicine* for happiness (subtype 2) increase consumers’ loneliness over time, whereas valuing possessions as a source of material *mirth* in life (subtype 3) correlates with reduced loneliness. Adopting a similar rationale, Gentina, Shrum and Lowrey [39] differentiates the acquisition- and sharing-based materialism. The former leads to increased loneliness (i.e., a vicious cycle), and the latter is associated with active coping actions and prosocial behaviours, which reduce loneliness (i.e., a virtuous cycle). To this end, an uncertain dynamic between consumers’ loneliness and nonhuman attachment seems to exist, which is worth further investigation.

### 3.2. Paradoxical Motivations of Affiliation and Self-Affirmation Exemplified in Product Selection (Blue Cluster)

The blue cluster contains three key themes, namely, *product selection*, *self-affirmation* and *prosocial behaviour*. This cluster extends consumers’ material attachment to the purchasing and consumption contexts, showing how consumers’ loneliness guides their product (including services and consumption experience) selection process. Although consumers use spending and consumption behaviours to strategically seek for social inclusion, as established in the previous cluster, literature related to the current cluster further suggests the paradoxical motivations of both *social affiliations* (i.e., to blend in) and *self-affirmation* (i.e., to stand out) that shape consumers’ purchase decisions [6,41,42].

Before dwelling on the major theme of this cluster (e.g., paradoxical motivations), we should acknowledge a small stream of less-relevant but interesting research that examines the aesthetic preferences of lonely consumers. Wang and Sirois [43] provide a good explanation in this regard by applying the conceptual metaphor theory. To illustrate, the theory suggests that individuals tend to metaphorise the higher-level abstract concepts (e.g., loneliness) into lower-level sensory perceptions (e.g., darkness and emptiness) [25]; hence, the metaphorical perceptions can be easily grasped to understand the higher-level abstractions [43]. Following this logic, consumers may avoid products that metaphorically reflect loneliness and prefer products that are associated with positive psychological states. For example, Wang and Sirois [43] find that lonely consumers prefer products presented with bright rather than dark lighting, as dark lighting is often associated with being excluded and isolated. Similarly, Su, Wan and Jiang [25] specified the metaphorical connection between visual emptiness and psychological emptiness. The authors suggested that the connection leads to lonely consumers’ preference for visual density regarding product design and package. The visual preference for density is also confirmed by Thomas and Saenger [7], who demonstrated that lonely consumers are more tolerant of over-crowded retail settings due to a feeling of relatedness associated with crowding environments. In addition, the loneliness-induced preferences are also found to vary due to individual differences (e.g., gender) [3,44] and cultural differences (e.g., independent and interdependent cultures) [45,46].

More pertinent to the current review study, the lonely consumers seem to face a paradox characterised by a motivation to conform to the social majorities while simultaneously affirming their unique identity as the ‘loner’ [41,42]. The paradox is evidenced by the conflicting findings reported in the empirical studies. For example, Guo, et al. [47] demonstrated that the socially excluded consumers are motivated to engage with green consumption as a prosocial behaviour to win social recognition. Lonely consumers are found to prefer ordinary products endorsed by the majority rather than distinct products [48]. Contrarily, other work has suggested that consumers disfavour the popular service options as they are inconsistent with their loneliness feelings [49]. Empirical evidence also indicates consumers’ preference for brands that can be used as a symbol to differentiate from rather than to affiliate with other consumers [50]. This is probably because consumers view their purchased products as an extended-self, and the lonely consumers incorporate their distinct selves into their purchase behaviours [3]. To this end, some researchers examined the phenomenon of solo consumers who prefer to always shop alone [2,26,27]. Although they may or may not experience loneliness, the solo consumers demonstrate some unique consumption patterns, such as variety seeking, brand consciousness and impulsiveness, which distinguish them from the majority ‘conformers.

Given the seemingly inconsistent findings, some studies have explored the boundary conditions that reconcile the paradox. Most notably, Lee and Shrum [6] proposed the differential needs hypothesis, which attributes the inconsistent findings to either efficacy or relation needs of lonely consumers. The hypothesis posits that loneliness impinges on the four fundamental human needs: control, meaningful existence, belongingness and self-esteem. The former two collectively produce the efficacy needs, and the latter two contribute to the relation needs. As a result, when consumers’ efficacy needs are threatened, they engage with conspicuous consumption to gain attention and show off their uniqueness. Meanwhile, when relation needs are violated, consumers demonstrate prosocial behaviours to reconnect and gain social acceptance. The differential needs hypothesis is also echoed by Su, et al. [51] study, which proposes a balancing mechanism between control restoration and belongingness maintenance in response to the differential needs of lonely consumers. In another study, researchers suggested that the differences in the consumption contexts (public or private) may also be a possible explanation. Consumers are found to choose minority-endorsed products to affirm their feelings of loneliness in private consumption, whereas they choose to conform to the majority when their choice is subject to public scrutiny [42]. In addition, Wan, Xu and Ding [41] emphasised consumers’ evaluations on their chances of successful re-affiliation: when the chance is high, consumers choose to signal their affiliation intention via their purchase decision while compromising their unique selves. By contrast, when the chance is low, consumers choose to diverge from the social majority and draw all resources to develop their distinct identities. Regardless of the underlying mechanisms, the identified literature collectively points to the complex loneliness-induced paradox, ‘to be or not to be unique’ [41], which is central to consumers’ behaviour responses in the consumption contexts.

### 3.3. Feelings- and Reasons-Based Information Processing Concerning Persuasions (Purple Cluster)

The purple cluster is a small cluster that is attached to the blue cluster. Containing two key themes, *feelings and reasons*, and *persuasions*, this cluster specifically examines the dual information processing mechanism (i.e., affect-based and cognition-based) by lonely consumers in response to commercial persuasions, such as advertisement appeals. By doing so, this cluster addresses persuasion strategies that influence the product choices of lonely consumers [52].

To illustrate, consumers engage with both affects and cognitions for the information process. Affects refer to feelings or emotions, and information processing based on affects is automatic and faster. Meanwhile, cognition involves reasons and logics, and information processing based on cognition is deliberate and consumes more cognitive resources [53]. For consumers who experience loneliness, our review suggests that loneliness impair individuals’ cognitive thinking, which makes affective persuasions more effective to emotionally vulnerable consumers. In other words, consumers tend to rely on feelings and emotions to process external information and, thus, the affect-based messages may be more effective in swaying lonely consumers’ product choices [53].

Such a view is shared and extended by Jiang, et al. [54]. Acknowledging the dual information processing mechanism, Jiang, Li, Li and Li [54] further explored the moderating effects of culture (individualism and collectivism) on lonely consumers’ responses to advertisement appeals. Individualistic consumers care more about their personal feelings, whereas consumers from the collectivism culture have a primary concern on justifying their decisions to social others. Consequently, the former consumer group is more responsive to persuasions that appeal to individual feelings, and the latter relies more on cognition-based reasoning [54]. In addition to cultural differences, consumers’ individual differences are also found to influence their responses to persuasions. For example, consumers who have a current orientation of time are more likely to cope with loneliness with active problem-solving, leading to cognition-based information processing; for future-oriented consumers, they tend to be more emotional and, thus, more responsive to affect-based appeals [55]. These findings may create implications for designing public communication strategies that promote prosocial behaviours among the lonely consumers in the post-pandemic era.

### 3.4. Consumer Well-Being in an Ageing Society (Red Cluster)

This cluster contains three key themes: *marketplace relationships*, *mobility and disability issues*, and *consumer well-being*. Being linked to the central theme of affiliation motivation, this cluster addresses lonely consumers’ compensatory attachment to marketplace relationships with salespersons, brand communities or online celebrities (e.g., parasocial and telepresence) [56,57,58]. A particular emphasis is placed on the well-being of the disadvantaged consumers or the elderly, who are vulnerable to loneliness and social isolation [59,60], especially given the current situation of COVID-19 [11,61].

The concept of marketplace relationships is critical to this cluster. It assumes that consumers who are socially excluded do not shun all relationships. Instead, they may switch to marketplace relationships that are built on monetary exchange and, hence, low in emotional investment [56]. Such a ‘relational domain switching’ applies especially to consumers who have an avoidance tendency or who are anxious towards interpersonal relationships [56]. Essentially, the marketplace contains more than business transactions. It is a venue enriched with social meanings, in which consumers and marketplace players participate to co-create consumption and relation values [62].

Literature in this regard has shown that consumers develop meaningful relationships with the brands, consumption communities, service providers and in-store salespersons that buffer interpersonal insecurity and compensate consumers’ fundamental need for belongingness [1,57,59]. For example, Snyder and Newman [57] showed that consumers treat brand communities as a viable avenue for social connections. The traditional communities, which are geographically-bound, are in decline in the fragmented modern society, whereas the brand communities are fast-growing on social media, which create social relatedness and ease loneliness [1]. In an offline setting, Smith, Rippé and Dubinsky [4] and Rippé, Smith and Dubinsky [5] investigated how the lonely consumers ‘befriend’ the in-store salespersons to cope with perceived deficiencies of social relationships.

Notably, Lim and Kim [60] examined an interesting pattern of marketplace relationships, termed as *parasocial*, whereby the media persona (e.g., social media celebrities) interacts with millions of audiences online, and each audience experiences the illusion of personal connectedness with the media persona. It is essentially a pseudo-intimate relationship in the virtual communities wherein the audiences develop a deep attachment. A similar phenomenon, *telepresence*, is also explored by one of the identified studies [59]. It describes a feeling of ‘being there’ when engaging with parasocial interactions in the technology-mediated environment. With telepresence, the actual physical environment fades away, and consumers are fully immersed in the parasocial experiences, which create a sense of assurance and belongingness that are much needed by the lonely consumers.

Both the works of Lim and Kim [60] (about parasocial) and Lim and Kim [59] (about telepresence) are conducted in the context of TV home shopping by old consumers, which highlight an important segment of lonely consumers that deserves special attention. In particular, the older consumers, especially retirees, ‘empty nesters’ or ‘solitary survivors’, are vulnerable to loneliness, yet they are often constrained by various age-related issues (e.g., mobility or disability) that prevent them from reaching out for social interactions [18,63]. To this end, some studies suggested that the multi-channel shopping technologies serve as an effective tool to facilitate the elderly and other disadvantaged consumer groups’ social participation process and, which enhances consumers’ happiness and well-being [17,62]. From the relatively old method of TV home shopping to the recent multi-channel and omnichannel commerce system, the elders can be empowered with the necessary resources to consume and to live independently, which is especially critical under the current context of COVID-19.

However, the harsh reality is that old consumers may have already been left out by the fast digitalisation in modern society, which causes a digital inequality between the young and old generations [64]. The shopping technologies may serve as assistance tools for the older consumers to meet their basic consumption needs while creating opportunities for social inclusion, yet an initial adoption barrier may exist for the elderlies with a low level of digital literacy. Indeed, the old consumers are deprived of digital capitals, such as skills and knowledge, that the younger consumers may take for granted. To a certain extent, there is an unbalanced diffusion of technologies. To illustrate, the innovative pioneers, often from the younger generation, are able to enjoy all the benefits of updated technologies, whereas the older consumers who are most in need of the technologies are often the last segment that the technologies penetrate into. As a result, the inequality marginalises the older consumers in the digital age, mirroring their social isolation within the communities [64]. Considering the context of COVID-19, the old consumers are said to be disproportionally jeopardised due to suffering higher health risks, having more difficulties using online technologies for services and information, and experiencing more serious social isolation and loneliness [11,61]. As emphasised by Xie, Charness, Fingerman, Kaye, Kim and Khurshid [11], when going digital becomes a necessity due to COVID-19, extra effort should be prioritised towards promoting technology penetration among the disadvantaged consumer groups.

### 3.5. Anthropomorphic Companionship in Virtual World (Lemon Cluster)

This cluster contains two key themes: *anthropomorphism* and *brand.* It is a small extension to the previous cluster, focussing on the marketplace relationships between lonely consumers and the brand communities with an emphasis on the anthropomorphic features of the virtual world.

Anthropomorphism refers to the tendency of consumers to attribute human-like features into nonhuman agents, which evoke consumers’ sympathy and feelings of relatedness [65,66]. It is often used in building online communities to encourage consumers to see human characteristics and seek connections. Examples of commonly incorporated anthropomorphic features include human-like brand mascots and brand logos with smiling faces or human-like motions [67]. The recent development of artificial intelligence has enabled more sophisticated human-like features to be introduced to the virtual world such as natural language processing and delicate facial expression. Although the lonely consumers look for compensatory social connections in the marketplace, the virtual brand communities increasingly provide human-like companionship that resembles the real-world social relationships.

Focusing on the virtual relationships between lonely consumers and anthropomorphic brands, Chen, Wan and Levy [67] found that consumers see the brand as a life partner, especially when they attribute an internal cause of loneliness. Orth, et al. [68] suggested that the anthropomorphic features enhance consumers’ positive attitude and elicit deeper attachments towards the brand. However, some studies have raised concerns about consumers’ privacy when interacting with anthropomorphic communities, suggesting that lonely consumers may feel insecure and anxious due to the human-like characteristics [66]. To reconcile the contradictory findings, Feng [69] proposed an inverted U-shaped curve that illustrates the relationship between consumers’ affiliation intention and the degree of anthropomorphism. Nonetheless, the question remains as to whether the anthropomorphic companionships serve as an effective solution to loneliness or they are meaningless obsessions that crowd out real social interactions and reduce consumers’ well-being.

## 4. Entering the Post-Pandemic Era: Conclusions and Proposed Research Agenda

Considering the long-lasting impacts of COVID-19 coupled with the societal structural changes, the feeling of loneliness is expected to prevail in the post-pandemic era. It may become a dominant psychological state that shapes consumption behaviours, creating commercial and social implications. Thus, this study conducts a timely review on the concept of lonely consumers by synthesising the scattered studies on the impacts of loneliness and social isolation on consumer behaviours. Adopting an inductive approach, our review study identifies 74 key themes from the relevant literature, which are subsequently merged to form five major theme clusters that address psychological, commercial and social aspects of lonely consumers.

### 4.1. Coping with Psychological Loneliness with New Habits

The review findings suggest a fundamental coping mechanism of loneliness among consumers by seeking secondary attachment to nonhuman agents [34,39]. Driven by an innate need of affiliation, lonely consumers compensate for their lack of social relatedness by attaching emotionally to materials, possessions, virtual reality or any other nonhuman agents that are of symbolic meanings. However, although consumers find comfort and assurance in nonhuman agents, they may diverge further away from the real-quality social interactions, which in turn increases consumers’ loneliness. Herein, a vicious cycle that leads to increasingly lonelier consumers seems to exist [34,38].

Applying the insights to the context of COVID-19, we are interested to see how the pandemic drives the trend of social isolation and creates profound impacts on consumers’ loneliness. In particular, future research may focus on consumers’ coping mechanisms in response to the pandemic-induced loneliness that is normalised as new habits in the post-pandemic era, and more importantly, how these coping mechanisms influence consumers’ loneliness reciprocally. For example, consumers are found to turn to communication technologies to keep in touch with important friends and families. Although the use of communication technologies is not a recent phenomenon, COVID-19 creates an unprecedented situation wherein technologies become the only available medium of communications. As a result, consumers may develop a reliance on technology-mediated communications that discourage them from real social interactions in the post-pandemic era. Given the above rationale, we propose the following specific research questions:

- To what extent does COVID-19 drive consumers’ adoption of in-door activities (e.g., reading, cooking, gardening and using social media), and to what extent do consumers attach to these activities to cope with loneliness during COVID-19?
- What activities ‘die’ with COVID-19 and ‘survive’ and become the new habits in the post-pandemic era? How are these two types of activities characterised?
- How do new habits impact consumers’ loneliness when social distancing is no longer practised? Does a virtuous or vicious cycle exist between the new habits and consumers’ loneliness?
- Comparing the pre- and post-pandemic situations at a macro-level, do the new habits reduce or increase the loneliness level of the general population?
- How do the new habits impact on consumers’ feelings of fear, uncertainty, confusion and distrust in official discourses associated with the pandemic-induced loneliness? Comparing the before and after pandemic situations at a macro level, do the new habits decrease or increase the negative feelings associated with consumers’ loneliness?

### 4.2. Changing Market Segments of Loners and Conformers

Our review study reveals the paradoxical motivations of self-affirmation and affiliation by the lonely consumers whereby they aspire to demonstrate the unique selves while conforming to the social majority [42]. This paradox creates commercial implications regarding the purchase decisions of lonely consumers as the purchased products are strategically used to demonstrate the consumers’ identities [33]. For example, purchasing majority-endorsed products or services often sends a social signal of affiliation, whereas choosing products or services that are appreciated only by the minorities reaffirms the consumers’ unique self-identity.

In the post-pandemic era, whether the consumption activities are driven by lonely consumers’ primary desire to conform or to differentiate remains to be seen. After the prolonged period (nearly a year to date) of social isolation due to COVID-19, it is unclear if the exclusion gives rise to a group of ‘loners’ who prefer uniqueness in their consumption or a group of ‘conformers’ who favour consensus and popular choices. To this end, our identified literature suggests an affect-based persuasion strategy that may appeal to the vulnerable emotions of lonely consumers [53]. The persuasion strategy is also found to be more effective in individualistic cultures compared with the collectivist culture. In addition to cultural differences, consumers’ age may also play an important role in consumers’ response to persuasion messages. Compared to working adults, consumers’ who are adolescences or retired elderlies are likely to be responsive to emotionally-appealing persuasions. While less discussed in the extant literature, age may serve as a critical moderator that influences the lonely consumers’ responses to external messages. Therefore, for future research, it would be interesting to examine the consumption patterns of lonely consumers in face of the paradoxical motivations after the pandemic-induced social isolation. In addition, how to design persuasive messages that effectively sway consumers’ consumption behaviours to create prosocial outcomes would be another worthwhile attempt. Thus, we propose the following research questions:

- To what extent do lonely consumers demonstrate the characteristics of ‘loners’ (‘conformer’) in the post-pandemic era?
- What are the consumer segments and how large are the segments if the market of lonely consumers is segmented using the characteristics identified in the above question? In addition to ‘loners’ and ‘conformers’, what are the other segments that may demonstrate the mixed characteristics?
- To what extent does the pandemic lead to a shrunk/enlarged segment of ‘loners’/’conformers’?
- Considering different cultural contexts, how should the persuasion messages (e.g., cognition- or affect-based) be designed for commercial or social purposes leading to desirable outcomes (e.g., to promote healthy food and COVID-19 vaccine)?
- Comparing lonely consumers belonging to different age groups, that is adolescences, working adults and retired elderlies, what are the most effective persuasion strategies (cognition- or affect-based) that lead to behavioural changes for commercial or social purposes?

### 4.3. Empowering the Disadvantaged with Technologies

The findings of the review suggest a unique relational domain switch where the lonely consumers replace social interactions with marketplace relationships with salespersons, online consumption communities or even virtual companions with anthropomorphic features [30,56]. Although such marketplace relationships may not be the ultimate solution to consumers’ loneliness, they may be especially helpful to the elderly who are ‘empty nesters’ or ‘sole survivors’ and to those who have mobility or disability issues. However, these socially disadvantaged consumers are often those who are left behind in the digital age, preventing them from reaching out using digital tools. We concur with Darcy, Yerbury and Maxwell [64] that digital inequality leads to a social divide that further marginalised the socially disadvantaged consumers.

Indeed, the socially disadvantaged consumers are heavily impacted by the pandemic due to their low level of digital literacy, yet the pandemic also creates a golden opportunity that empowers the disadvantaged with digital technologies. During the pandemic, an expedited trend of digitalisation has been witnessed worldwide even among the technologically challenged population. Future research efforts may be directed to investigate the technology diffusion trends within this ‘laggard’ segment due to the catalytic effect of COVID-19. More importantly, we are interested to see the associated changes in consumers’ well-being when empowered with the technologies that connect them with the wider world. Therefore, we propose the following research questions for further investigation:

- To what extent does the lack of digital resources (e.g., skills and knowledge) create social isolation and loneliness among the disadvantaged consumers?
- To what extent does COVID-19 contribute to the diffusion of digital technologies among the disadvantaged consumers?
- To what extent do the anthropomorphic products (e.g., service robots) replace consumers’ needs for social interactions, especially for the disadvantaged consumers?
- How should resources be coordinated to enhance the well-being (e.g., informational, physical health and mental health) of the disadvantaged consumers?

The review study is not without limitations. Firstly, we include only journal articles in the review list. Although the purpose is to ensure data quality, the exclusion of a considerable number of conference articles and book chapters restricts the comprehensiveness of the review study. Secondly, the review findings including the identified key themes and the thematic clusters are based on the authors’ subjective evaluations of the journal articles. Thus, we claim this research as a personal interpretation of extant literature on the lonely consumers. Finally, although socio-demographic factors, such as gender, age and living environment, are identified as a key theme from the extant literature, their impact on lonely consumers are not consistently discussed in the review findings. This is due to the fact that socio-demographic factors were examined from diverse perspectives in a piecemeal manner, which is difficult to draw conclusive findings. For example, Mittal and Silvera [3] held gender as a predictor of material versus experiential consumptions, whereas O Sullivan and Richardson [1] examined the effect of feminism in consumption communities to cope with loneliness. Lim and Kim [59] found that lonely older consumers are motivated to shop from multiple channels, while other studies suggested that adolescences also utilise both online and offline shopping to seek social inclusion. To this end, we suggest that the issue associated with socio-demographic differences remains as a worthwhile direction of consumption literature. Future scholars may direct their attention to this aspect.

## Figures and Tables

**Table 1:** List of key themes and descriptions.

Key Theme *	Brief Description	Occurrence
Production selection	The impacts of perceived loneliness or social isolation on consumers’ choice or preference of products or services	12
Brand	Response to brand communities, such as brand attitude and brand participation	9
Affiliation motivation	Fundamental motivation of belongingness to a social group, which guides lonely consumers’ consumption behaviours	8
Attachment to nonhuman	Attachment to materials or products as a replacement of interpersonal relationships	8
Self-affirmation	Confirmation and enhancement of self-identity and personal uniqueness, which guide lonely consumers’ consumption behaviours	7
Anthropomorphism	Built-in human-like features in nonhuman agents, appealing to lonely consumers’ empathy and liking	6
Marketplace relationships	Relationships with an in-store salesperson, hosts of e-commerce sites or brands due to lack of quality social interactions in consumers’ daily life	6
Mobility and disability issues	Physiological restrictions that cause loneliness and social isolation, which are often associated with senior consumers	5
Persuasion	External cues such as advertisements and promotional information that appeal to consumers’ loneliness, and the associated responses from consumers	5
Prosocial behaviour	Behaviours for social well-being, such as helping others, donation and green consumption, which are demonstrated by lonely consumers to gain social inclusion	5
Consumer well-being	Physical and mental conditions of consumers, which are often impaired by loneliness but improved via several coping strategies	4
Distinct and popular products	Preferences for minority- or majority-endorsed products, depending on consumers’ motivations and needs to cope with loneliness	4
Cultural effects	Macro-level cultural impacts on lonely consumers, such as collectivism vs. individualism, independent vs. interdependence, low-context vs. high-context	3
Experiential and material products	Preferences for experiential or material consumption by lonely consumers	3
Feelings and reasons	Information processing mechanisms, which can be affect-based or cognition-based in response to persuasions	3
Gender differences	Gender as a moderator of various behaviours of lonely shoppers	3
Materialism	Importance attached to owning symbolic material possessions, which is not only the cause but also a consequence of loneliness	3
Reciprocal effect of loneliness	Consequences of loneliness, which in turn reinforce loneliness, which depicts the dynamic impacts of loneliness, such as materialism	3
Solo shopper	Consumers who engage with consumption activities alone, who may or may not be lonely	3
Visual preferences	Aesthetic preferences, which may be modified by consumers’ state of loneliness, such as a preference for warmth and crowdedness	3

* Only parts of the themes are used for generating co-occurrence network based on their association strength.

## Data Availability

Data sharing not applicable.

## Appendix A

**Source**	**Key Point**	**Key Theme**	**Assigned Cluster ***
Chen, Wan and Levy [67]	Socially excluded consumers show preferences for anthropomorphised brands in seeking for affiliation; the resulted band-consumer relationship is influenced by consumers’ attributions of causes of social isolation.	Anthropomorphism; Brand; Marketplace relationships; Internal and external attribution; Affiliation motivation	Commercial implications
Darcy, Yerbury and Maxwell [64]	Mobile technologies work as assistant technology to support senior people with disability.	Assistant technology; Digital inequality; Mobility and disability issues	Social implications
Das, Echambadi, McCardle and Luckett [31]	Lonely consumers are more likely to use the web for information searching.	Personality	Psychological implications
David and Roberts [38]	Phone snubbed consumers experience social isolation which leads them to social media to regain a sense of inclusion.	Phubbed; Attachment to nonhuman; Reciprocal effect of loneliness	Psychological implications
Dennis, Alamanos, Papagiannidis and Bourlakis [17]	Multi-channel shopping increases the happiness and well-being of socially excluded consumers, such as consumers with mobility/disability issues.	Multi-channel shopping; Mobility and Disability issues; Consumer well-being	Social implications
Dennis, Bourlakis, Alamanos, Papagiannidis and Brakus [62]	social isolation leads consumers to participate in multi-channel shopping, which co-create values for consumers and shopping channels.	Value co-creation; Multi-channel shopping; Mobility and disability issues; Consumer well-being	Social implications
Donthu and Gilliland [26]	Single shoppers by choice or by circumstance demonstrate some unique shopping styles to compensate the feeling of loneliness, including variety seeking, risk aversion, price consciousness, brand consciousness, impulsiveness and TV watching.	Solo shopper; Brand; Impulsive or compulsive buying; Risk-taking	Commercial implications
Duclos, Wan and Jiang [37]	social isolation increases the instrumental value of money which leads consumers to make riskier financial decision	Instrumentality of money; Risk-taking; Gambling	Psychological implications
Feng [69]	Loneliness influences consumers’ responses to anthropomorphic products; product types play a role as well.	Product Selection; Anthropomorphism; Utilitarian and hedonic products	Commercial implications
Fitch [24]	The accessibility of local food and retail stores is assessed for socially excluded consumers	Retail reform; Accessibility; Mobility and disability issues	Social implications
Gao and Mattila [49]	Socially excluded consumers favour distinctive (vs. popular) green hotels due to self-affirmation motivation.	Self-affirmation; Green consumption; Prosocial behaviour; Product selection; Distinctive and popular products	Commercial implications
Gentina, Shrum and Lowrey [39]	Loneliness leads to passive and active coping strategies associated with different types of materialism, which increases or decreases unethical behaviours among young consumers	Materialism; Unethical behaviours	Psychological implications
Goodwin and Lockshin [27]	The concept of solo shoppers is proposed and differentiated from lonely shoppers.	Solo shopper; Stereotypes	Commercial implications
Guèvremont and Grohmann [70]	Socially excluded consumers are more emotionally attached to authentic brand in seeking for belongingness	Brand; Attachment to nonhuman; Self-affirmation	Commercial implications
Guo, Zhang, Liao and Wu [47]	Socially excluded consumers engage with green consumption as a signal to gain social inclusion.	Green consumption; Prosocial behaviour; Costly signalling; Self-sacrifice	Commercial implications
Hart and Royne [65]	Loneliness enhances consumers’ responses to anthropomorphism appeals in the case of advertising messages, leading to a more positive attitude toward the brand	Anthropomorphism; Brand; Persuasion	Commercial implications
Her and Seo [2]	Anticipated loneliness reduces consumers’ intention to dine alone in public restaurant.	Sole shopper;	Commercial implications
Hwang and Mattila [44]	social isolation causes negative emotions (e.g., loneliness) and a sense of losing control which influences consumers’ attitude toward the brand. The relationships are more salient among female consumers.	Gender; Brand;	Commercial implications
Jiang, Li, Li and Li [54]	Cultural influences (individualism and collectivism) moderate socially excluded consumers’ responses to external persuasions; Consumers under individualism (collectivism) culture are more responsive to feelings- (reasons-)based advertisement.	Culture; Individualism and collectivism; Persuasion; Feelings and reasons;	Commercial implications
Jiang, et al. [71]	Socially excluded consumers who have a present- (future-) orientation tend to exert less (more) self-regulation.	Self-regulation; Time orientation	Social implications
Kemp, Moore and Cowart [52]	Lonely consumers respond more favourably to self-referent advertising appeals	Persuasion; Self-affirmation	Commercial implications
Kim and Jang [18]	Lonely consumers tend to cope with loneliness with experiential consumptions such as dining out, travel and drink. Age differences are explored.	Experiential consumption	Social implications
Kim, Kang and Kim [63]	Loneliness leads to older consumers’ mall shopping motivation which can be consumption-related and experiential-related.	Shopping motivation; Experiential and material products	Social implications
Kim, Kim and Kang [58]	Young consumers’ loneliness motivates their media use and shopping behaviour	Shopping motivation	Social implications
Lastovicka and Sirianni [40]	Loneliness enhances consumers’ love of material possession	Materialism; Attachment to nonhuman; Consumer well-being	Psychological implications
Lee and Shrum [6]	Social isolation threatens the differential needs of consumers; the threatened efficacy needs (explicitly rejected) triggers conspicuous consumption, whereas the threatened relational needs (implicitly ignored) triggers prosocial behaviours.	Prosocial behaviour; Conspicuous consumption; Differential needs hypothesis; Self-affirmation; Affiliation motivation	Commercial implications
Lee, Shrum and Yi [45]	Consumers’ behavioural responses to social isolation are dependent on the cultural norm where the exclusion is communicated; norm-congruent communications lead to prosocial behaviour and counter-normative communications lead to conspicuous consumption	Prosocial behaviour; Conspicuous consumption; Differential needs hypothesis; Culture; High-context and low-context communications	Commercial implications
Lim and Kim [60]	Older consumers’ loneliness leads to their parasocial interactions with TV host, which increase their purchasing intention of TV home shopping.	TV home shopping; Parasocial interaction; Mobility and disability issues; Marketplace relationships	Social implications
Lim and Kim [59]	Older consumers’ loneliness motivates their TV home shopping behaviour, which creates a sense of telepresence and leads to consumers’ satisfaction with TV shopping	TV home shopping; Telepresence; Shopping motivation	Social implications
Loughran Dommer, Swaminathan and Ahluwalia [50]	Socially excluded consumers use a brand to differentiate themselves horizontally and vertically; Low self-esteem consumers increase perceptions of group heterogeneity (seek to protect their future belongingness) and subsequently increase their attachment to horizontal (vertical) brands	Brand; Attachment to nonhuman; Affiliation motivation	Commercial implications
Lu and Sinha [53]	Social isolation impairs consumers’ cognitive thinking, which makes them more responsive to affect-based persuasion.	Feelings and reasons; Persuasion	Commercial implications
Mead, Baumeister, Stillman, Rawn and Vohs [33]	Socially excluded consumers use spending and consumption to seek affiliation, where they sacrifice personal and financial well-being for the sake of social well-being.	Affiliation motivation; Spending and consumption; Self-sacrifice	Psychological implications
Mittal and Silvera [3]	Loneliness and gender predict consumers’ attachment to purchases (material and experiential), which explain future purchase intentions.	Extended self; Attachment to nonhuman; Gender; Product selection; Experiential and material products	Commercial implications
Mourey, Olson and Yoon [30]	Consumers attach to anthropomorphic products as an alternative to interpersonal interactions to gain social assurance.	Anthropomorphism; Attachment to nonhuman; Prosocial behaviour; Product selection; Social assurance	Psychological implications
O Sullivan and Richardson [1]	Consumption communities function as a self-help group which provides ‘treatment’ for loneliness.	Community; Gender	Social implications
Orth, Cornwell, Ohlhoff and Naber [68]	Consumers’ loneliness and anthropomorphism tendency moderate their responses to brand visual, leading to brand liking	Brand; Anthropomorphism; Persuasion	Commercial implications
Pieters [34]	Loneliness contributes to materialism, and materialism reinforces consumers’ loneliness to a weaker extent depending on the subtypes of materialism.	Materialism; Reciprocal effect of loneliness; Attachment to nonhuman	Psychological implications
Rippé, Smith and Dubinsky [5]	Loneliness (social and emotional) contributes to consumers’ interaction with in-store salesperson, which leads to purchase intention and retail patronage	In-store interaction; Marketplace relationships	Social implications
Smith, Rippé and Dubinsky [4]	Loneliness (social, emotional and physical isolation) leads to enjoyment of in-store interaction with salespersons.	In-store interaction; Marketplace relationships	Social implications
Snyder and Newman [57]	Lonely consumers express a higher intention to join brand communities in seek for belongingness.	Brand; Community; Marketplace relationships; Affiliation motivation; Consumer well-being	Social implications
Su, Jiang, Chen, DeWall, Dahl and Lee [51]	Social isolation threatens consumers’ sense of control. The motivation of control restoration and belongingness maintenance influence their switching behaviour.	Switching behaviour; Control restoration; Affiliation motivation	Commercial implications
Su, Wan and Jiang [25]	Social isolation causes consumers’ psychological emptiness, which enhances their preference for visual density.	Visual preference; Density; Psychological emptiness; Product selection	Commercial implications
Suresh and Biswas [32]	Loneliness is posited as an antecedent factor to internet addiction which leads to compulsive buying online among young consumers	Impulsive or compulsive buying; Attachment to nonhuman; Internet addiction	Psychological implications
Tan and Hair [35]	Loneliness triggers consumers’ ethnocentrism which leads favourable responses to domestic products; the ethnocentrism reinforces consumers’ loneliness	Product selection; Domestic and foreign products; Ethnocentrism; Reciprocal effect of loneliness	Psychological implications
Thomas and Saenger [7]	Social isolation increases consumers’ motivation to affiliate which modifies their perception of crowded retailing setting and associated shopping behaviour	Visual preference; Affiliation motivation; Crowdedness	Commercial implications
Wan, Xu and Ding [41]	Stable social isolation leads to consumers’ preference for distinctive products due to the self-affirmative motivation.	Product selection; Distinctive and popular products; Self-affirmation; Stable or unstable exclusion	Commercial implications
Wang, Zhu and Shiv [42]	Lonely consumers are more likely to choose minority-endorsed product to maintain their feelings of loneliness, but they choose to conform with the majority when their choice is under public context.	Self-affirmation; Product selection; Affiliation motivation; Distinctive and popular products	Commercial implications
Wang and Lalwani [36]	Culture (independent and interdependent) moderates consumers’ impression management pursuit due to social isolation; interdependent consumers tend to give up impression management when socially excluded.	Impression management; Culture; Independent and interdependent; In-group identification	Psychological implications
Wang and Sirois [43]	Loneliness influences consumers’ visual preference of product, i.e., warmer products are favoured over darker products	Product selection; Visual preference; Warmer and darker products	Commercial implications
Wei [61]	Introversion predicts more severe loneliness due to social distancing during COVID-19.	Social distancing; Personality; COVID-19	Social implications
Whelan, Johnson, Marshall and Thomson [56]	Consumers with interpersonal insecurity turn to marketplace relationships (with brands and salespersons) as a compensation strategy.	Brand; Marketplace relationships; Anxiety and avoidance	Social implications
Xie, Charness, Fingerman, Kaye, Kim and Khurshid [11]	Older consumers are disproportionately affected by the COVID-19 pandemic, which requires solutions	Covid-19; Social distancing;	Social implications
Xie, Chen and Guo [66]	Social isolation moderates consumers’ privacy concern when using anthropomorphic online services and subsequent purchase behaviour.	Anthropomorphism; Privacy	Commercial implications
Xu and Jin [55]	When consumers are socially excluded, they are more likely to have a problem-solving tendency choose utilitarian products. By contrast, when consumers imagine being socially excluded in the future, they are more likely to use emotions to solve problems and choose hedonic products.	Product selection; Utilitarian and hedonic products; Time orientation; Feelings and reasons	Commercial implications
Yang, Yu, Wu and Qi [46]	Socially excluded consumers prefer experiential purchases over material to fulfil relational needs. The effect is stronger among consumers with interdependent self-construal.	Product selection; Experiential and material products; Independent and interdependent; Self-construal	Commercial implications
Yi, Kim and Hwang [48]	Socially excluded consumers tend to choose ordinary products to restore threatened self-concept.	Product selection; Distinctive and popular products	Commercial implications
* Cell colours indicate the assigned clusters of the respective papers in the co-occurrence network.			
